# Facing COVID-19 in Liberia: Adaptations of the Resilient and Responsive Health Systems Initiative

**DOI:** 10.5334/aogh.3245

**Published:** 2021-10-08

**Authors:** Regan H. Marsh, Chelsea Plyler, Mary Miller, Robin Klar, Mukhtar Adeiza, Ian Wachekwa, Freda Koomson, James Luke Garlo, Kebeh Kruah, Sodey C. Lake, Rita Matte, Rebecca Cook, Daniel Maweu, Lila Kerr, Onyema Ogbuagu, Kristina Talbert-Slagle, Bernice Dahn

**Affiliations:** 1 Brigham and Women’s Hospital, Partners In Health, US; 2 Yale School of Medicine, US; 3 New York University Rory Meyers College of Nursing, US; 4 Yale School of Medicine, John F. Kennedy Medical Center, US; 5 John F. Kennedy Medical Center, LR; 6 Yale School of Medicine, University of Liberia, Redemption Hospital, US; 7 Partners In Health, Mass General Hospital/Harvard Medical School, LR; 8 Partners In Health-Liberia, LR; 9 University of Liberia College of Health Sciences, LR

## Abstract

The 5-year Resilient and Responsive Health Systems (RRHS)-Liberia Initiative, funded by PEPFAR via HRSA, launched in 2017 and was designed to support the implementation of Liberia’s National Health Workforce Program as a means to improving HIV-related health outcomes. The COVID-19 pandemic, arrived in Liberia just five years after Ebola and during RRHS-Liberia’s fourth year, impacted educational programs and threatened the project’s continued work. This paper presents the challenges that the COVID-19 pandemic posed to the RRHS partners, as well as adaptations they made to maintain progress towards project goals: 1) contributing to Liberia’s 95-95-95 HIV targets via direct service delivery, and 2) building a resilient and responsive health workforce in Liberia via instruction and training. Direct health service impacts included decreased patient volumes and understaffing; adaptations included development of and trainings on safety protocols, provision of telehealth services, and community health worker involvement. Instruction and training impacts included suspension of in-person teaching and learning; adaptations included utilization of multiple online learning and virtual conferencing tools, and increasing clinical didactics in lieu of bedside mentorship. The RRHS team recommends that these adaptations be continued with significant investment in technology, IT support, and training, as well as close coordination among partner institutions. Ultimately, the RRHS Liberia consortium and its partners made significant strides in response to ensuring ongoing education during the pandemic, an experience that will inform continued service delivery, teaching, and learning in Liberia.

## Background

The 5-year Resilient and Responsive Health Systems (RRHS) Liberia launched on January 1, 2017 and was designed to support Liberia’s national Health Workforce Program as a means to improving health outcomes [1], particularly the country’s performance against the 95-95-95 targets for HIV. RRHS-Liberia is implemented by a consortium of US academic and nongovernmental organizations that work in partnership with Liberian health facilities and training institutions in Montserrado County—Liberia’s population center and home to the highest-burden HIV clinics—and Maryland County, a rural region that serves as a model for universal health care. The RRHS-Liberia consortium has implemented a combination of pre-service training, in-service training, and direct service delivery to enable both short- and long-term improvements in HIV outcomes and to strengthen Liberia’s health workforce.

On March 16, 2020, when the first case of COVID-19 was confirmed in Liberia, RRHS-Liberia was at the start of its fourth of five years. As of June 10, 2021, Liberia has had 2,484 confirmed cases of COVID-19 with 93 deaths [2]. This paper presents the impact of the COVID-19 pandemic on RRHS-Liberia as well as the adaptations made to maintain progress towards project goals: 1) contributing to Liberia’s 95-95-95 HIV targets via direct service delivery, and 2) building a resilient and responsive health workforce in Liberia via instruction and training.

## Direct Service Delivery: Impact of COVID-19

COVID-19 led to a decrease in routine service delivery and utilization at all four clinical sites supported by RRHS-Liberia: John F. Kennedy Medical Center (JFKMC) and Redemption Hospital (RDH) in Montserrado County, and JJ Dossen Hospital and Pleebo Health Center (Pleebo HC) in Maryland County. On April 10, 2020, the Government of Liberia imposed a three-week 3pm-to-dawn curfew to curtail spread of the virus; the curfew was then revised to 6pm, then 9pm, until it was lifted on July 22. The curfew also affected public transportation and caused widespread loss of livelihood; as a result, patients and clinical staff struggled to travel to facilities for both logistic and financial reasons. Patients also expressed concern about exposure to COVID-19 at hospitals, resulting in decreased utilization. At JFKMC, the number of patients seeking HIV services dropped by more than 50% (internal data). In Maryland County, general outpatient clinics saw a pronounced decline in utilization.

In the context of the deaths of health workers during the 2014–15 Ebola outbreak, staff at RDH and JFKMC feared nosocomial exposure, and absenteeism rose. At JFKMC, staff 60 years and older and those with comorbid conditions were exempted from duty until June 21, 2020. Staff who were exposed to COVID-19 were excused from clinical services to quarantine. Resident physicians went on strike to voice their safety concerns. As a result, the hospitals were consistently understaffed.

At the start of the pandemic, chronic non-communicable disease (NCD) care clinics at JFKMC were suspended and at JJ Dossen, and Pleebo HC numbers of NCD visits/day were reduced and patients given multimonth dispensing. JJ Dossen Hospital and Pleebo HC temporarily cancelled elective surgery and gynecology clinics. At RDH, HIV and syphilis testing was halted. Clinical care was further interrupted following each reported COVID-19 case; in the initial stages of the pandemic, wards with COVID-19 exposure were closed for up to four weeks.

## Direct Service Delivery: Adaptations

### Safety protocols

Over time, clinicians employed by by RRHS-Liberia assisted Liberian facility leadership in establishing safety protocols and trained staff. RRHS clinicians helped to develop national guidelines on disinfection and reopening following COVID-19 cases, resulting in shorter shutdowns following each reported COVID-19 case. RRHS clinicians at JFKMC and JJ Dossen Hospital trained staff on COVID-19 IPC measures, implemented clinical screenings and triage protocols at hospital entry points, made masks mandatory, and allowed emergency room care only after screening. At all facilities, personal protective equipment (PPE) and hand hygiene materials were scarce, resulting in repeated use. RRHS partners procured PPE and Infection Prevention Control (IPC) supplies for facilities.

Adaptations were largely successful, and patient volumes increased at all clinical partner sites. As health and safety protocols were clarified and enforced, staff and patients gained confidence in care delivery. One notable example of success was in HIV care: the RRHS team at JFKMC designed and implemented a protocol specific to the Infectious Disease Clinic (IDC), enhancing ventilation in clinic spaces, and creating extra waiting space. IDC staff provided anti-retroviral (ARV) prescriptions for three-month periods in order to prevent treatment interruptions. With these adaptations, there were no recorded COVID-19 cases amongst patients or staff at the IDC, and HIV service delivery continued uninterrupted. Although HIV testing and viral load testing volumes dropped precipitously at the beginning of the pandemic, the success of these adaptations resulted in marked improvement from July onward, ultimately exceeding pre-pandemic levels.

### Telehealth

Given limitations in technology, IDC, JJ Dossen, and Pleebo Health center staff used their personal devices to make phone calls to follow up with patients newly initiated on ART. This resulted in ability to successfully connect with patients, while protecting patients and staff from COVID-19 exposure.

### Community Health Worker Involvement

In Maryland County, community health workers (CHWs) played a critical role in ensuring continuity of care for patients with chronic conditions such as HIV, mental health disorders, and other NCDs. CHWs also helped clinics transition to multi-month dispensing of medications. CHWs led active community-based tracing for those lost to follow-up, and patients were successfully brought back to care after a high number of missed appointments in the early months of the pandemic.

## Health Services Instruction and Training: Impact of COVID-19

All RRHS-Liberia teaching and mentoring activities were affected by the pandemic. At the University of Liberia (UL), undergraduate medical students, as well as nursing and midwifery students, were unable to attend classes in person. The Certificate in Health Systems Leadership and Management, an executive education program, was postponed during the peak of the pandemic.

At Tubman University, clinical rotations were officially canceled for nursing students from March – July 2020, and graduation was delayed by six months. As a result, the licensing exam could not be offered as students who were due to graduate in July 2020 did not yet have a university diploma, delaying official hiring and deployment of graduate nurses.

Physician graduate medical education continued both at JFKMC and J.J. Dossen. However, didactic classes at JFKMC were temporarily discontinued due to a lack of lecture halls compatible with social distancing guidelines. Fear of patient contact among faculty and trainees disrupted clinical training, with reductions in many clinical procedures. At JJ Dossen, due to cancellation of elective surgeries, trainees received less procedural experience.

Many conferences and Continuing Professional Development (CPD) opportunities were canceled. Some moved online, posing a challenge in rural Maryland County, where many hospital and clinic staff have limited internet and IT infrastructure. Training on new national HIV management guidelines was delayed.

## Health Services Instruction and Training: Adaptations

### Clinical exposure

RRHS partners made a number of adaptations to facilitate continued clinical learning for trainees. While formal rotations for nurse trainees at Tubman University were cancelled and graduation delayed, many graduate nurses volunteered on the wards alongside clinical preceptors. RRHS partners coordinated with Tubman University and Maryland County Health Team to train nursing students on IPC and case management, so that nursing students who volunteered on the wards were more equipped to stay safe while gaining continued clinical experience. At J.J. Dossen, safety precautions and the small group size of physician trainees enabled continuation of in-person morning reports, small teaching sessions, and bedside rounds. Despite decreased elective procedures, resident trainees were able to continue learning through managing emergency cases. Educators at J.J. Dossen further adapted by increasing clinical didactics and skills workshops.

### Online learning

#### Medical residents

At JFKMC, all didactic lectures, morning reports, journal club, seminars, and presentations resumed via videoconference. RRHS team members enlisted an IT officer to run and monitor the videoconference platform, which was crucial to the success of its use. These adaptations allowed for residents to continue progressing through the training program with success. In fall 2020, Internal Medicine residents took the Liberian College of Physicians and Surgeons exit exams and achieved a 100% pass rate; 60% of residents also passed the regional West African College of Physicians exams. At JJ Dossen, Zoom conferences were held with family medicine residency colleagues in Monrovia. The family medicine residents took the Liberian College of Physicians and Surgeons exit exam with 100% pass rate, utilizing Zoom during the oral aspects of the LCPS exams to facilitate faculty from both Harper and Monrovia participating as examiners.

#### Nursing students

UL/Tubman National Institute of Medical Arts (TNIMA) nursing students utilized online classes through the MOODLE online learning platform, with set up and training facilitated by UL. RRHS partners established WhatsApp platforms for students, faculty, and affiliated hospital staff and used to disseminate COVID-19 related messages and guidelines, lessons learned from webinars, and standard operating procedures.

#### Medical students

Prior to the COVID-19 pandemic, with RRHS funding, all AMD students were registered to use Lecturio, an online learning platform, with a customized portal mapping the platform’s digital content onto AMD’s existing curriculum. Lecturio also allows students to download videos and other content for viewing and use offline via a custom smartphone application. While in-person classes were suspended, Lecturio became the students main means of instruction and study. As there was an existing high level of institutional support, medical faculty and students were able to shift quickly to remote instruction using this platform. As of June 2021, 297 active AMD users have accessed Lecturio’s remote learning materials. Preclinical students comprised the majority of student learners on Lecturio, watching 775 individual learning videos.

#### Certificate in Health Systems Leadership and Management students

The UL Certificate in Health Systems Leadership and Management resumed in August following postponement in June. Students who lived in Monrovia convened for in-person class with masks and social distancing in a large well-ventilated auditorium. WhatsApp and Zoom tools were leveraged for ongoing mentorship for remote presentations by participants from Maryland County.

Despite the success of online learning across RRHS-Liberia initiatives, significant challenges arose. The UL/TNIMA program experienced some difficulty navigating online learning, as some students and instructors did not have laptops, email addresses, or funds to buy cellular data. Students in the TNIMA diploma program could not access online learning, as the school lacks strong internet connectivity. As a result, students in the program did not return to classes until January 2021. While not equally, these challenges affected all cadres of trainees, with some resident physicians also missing virtual meetings and trainings due to lack of necessary devices, cellular data credit, and/or bandwidth.

### Virtual conferencing

#### Medical curriculum reform

Importantly, ongoing work on curriculum reform for AMD became a focal point during the pandemic. A Technical Working Group of AMD faculty and administration, supported by international partners, met weekly via videoconferencing. Due to the slowdown and shift to remote work in some of their other responsibilities, faculty became more available to participate in Technical Working Group activities, strengthening the pace of this initiative. RRHS-Liberia paid for cellular data for participants.

#### CPD

Despite challenges in travel during COVID-19, practicing health professionals, particularly mid-level HIV and TB providers, were able to participate in CPD and global conferences. RRHS-Liberia redirected funding to support virtual conference participation to ensure success. RRHS partners in Maryland County made investments in IT and physical infrastructure, including a dedicated medical library for such meetings, a shared computer for trainee physicians, and an internet router. As a result, frontline providers in Maryland County successfully participated in three major global conferences. At JFKMC, resident education, including morning report, journal club, didactic lectures and other trainings were conducted over Zoom.

## Themes and Recommendations

These experiences give rise to several themes and recommendations. Flexibility and adaptability of in-country leadership proved crucial to successful adaptations to the COVID-19 pandemic, particularly in implementing online learning. Additionally, a culture of development and improvement at Liberian training and educational institutions facilitated swift adaptations.

Furthermore, the largely successful utilization of online learning across multiple training and educational institutions illustrates that with targeted investment, high quality online learning is possible in low resource settings. Importantly, conversion to virtual modalities, including both telehealth and online learning, required significant investment in technology, IT support, and training. In this case, online programs were more successful when adapted to the context, including making materials available while offline to accommodate unreliable internet access.

Close coordination between training institutions and leadership at health facilities hosting clinical trainees is necessary to ensure the safety of students on clinical rotations, by verifying availability of PPE, supervisors, and the existence of IPC procedures. Lastly, stock management and funding to ensure adequate supplies of PPE and IPC materials for students and trainees ensures that learners can safely continue their clinical education.

Liberia has to-date experienced a relatively low number of COVID-19 cases, with 2,484 total diagnosed cases as of June 10, 2021, although is now facing a third wave [2]. While the early burden of disease was not severe, these experiences and recommendations were generated to provide high levels of protection and continue to offer relevant solutions for the continuing COVID-19 pandemic and potential future crises.

## Conclusion

The COVID-19 pandemic forced the RRHS-Liberia consortium to explore and implement innovative methods previously considered challenging within the resource-constrained setting, including telehealth and online learning. Though difficulties accompanied these shifts, RRHS partners made significant strides in response to the pandemic that will inform the future of service delivery, teaching, and learning in Liberia. The limitations to the initial success of these adaptations provide insight into new opportunities for ongoing adaptation and continued investment in ensuring resilience and responsiveness in Liberia’s health system.
